# Supplementary material for: Context-defined cancer co-dependency mapping identifies a functional interplay between PRC2 and MLL-MEN1 complex in lymphoma
Source: Nat Commun. 2023 Jul 17;14:4259. doi: 10.1038/s41467-023-39990-5 (PMC10352330; doi:10.1038/s41467-023-39990-5)
Supplement: Supplementary file 1 — Supplementary Information [file 41467_2023_39990_MOESM1_ESM.pdf]

Supplementary Information

Context-defined cancer co-dependency mapping identifies a functional interplay between PRC2 and MLL-MEN1 complex in lymphoma

Xiao Chen, Yinglu Li, Fang Zhu, Xinjing Xu, Brian Estrella, Manuel A. Pazos II, John T. McGuire, Dimitris Karagiannis, Varun Sahu, Mustafo Mustafokulov, Claudio Scuoppo, Francisco J. Sánchez-Rivera, Yadira M. Soto-Feliciano, Alberto Ciccia, Laura Pasqualucci, Jennifer E. Amengual, Chao Lu

This file includes:

Supplementary Figures 1 to 10

Supplementary Fig. 1

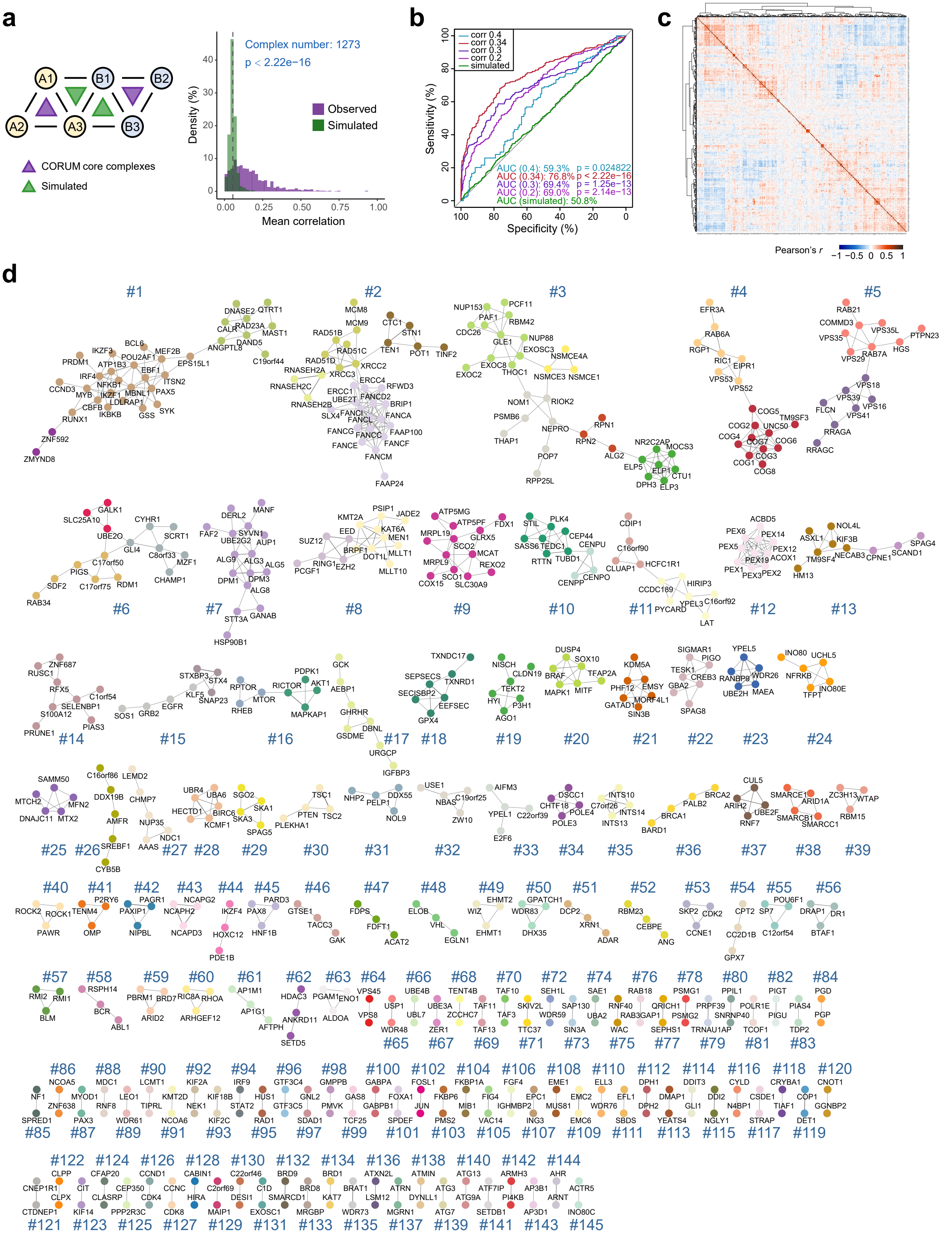

Supplementary Fig. 1: Dependency correlation network construction for non-essential genes, related to Figure 1.

a Left, a schematic diagram showing the interactions mapped to reported protein biochemical interactions between CORUM core complex members as an observed dataset (purple) and randomly selected interactions in equal number as a simulated dataset (green). In total, 1,273 CORUM human core complexes are observed and simulated. Right, histogram of correlation scores of the observed and simulated complex-level interaction datasets. The basal positive correlation score (0.055) is indicated as a dashed line. P value was determined by unpaired two-tailed Student's *t*-test; p < 2.22e-16.

b ROC plot showing the sensitivity and specificity of identifying complex-level interactions using different cutoffs. The AUC scores and corresponding p values (determined by Delong test) are indicated on the bottom right; p (corr0.4 vs. simulated) = 0.0248; p (corr0.34 vs. simulated) < 2.22e-16; p (corr0.34 vs. simulated) = 1.25e-13; p (corr0.34 vs. simulated) = 2.14e-13.

c Heatmap showing the dependency correlation matrix containing 590 genes that have at least one strong interaction with other genes (Pearson's r > 0.4). The correlation scores are colored from blue to red based on Pearson's r values from -1 to 1, respectively.

d Dependency correlation networks generated from the dependency correlation matrix using Genets (only top 15 strongest interactions for each node are shown). The 145 modules are numbered accordingly. The thickness of lines between genes indicates the correlation strength.

Source data are provided in Supplementary Data 2 and Supplementary Data 3.

Supplementary Fig. 2

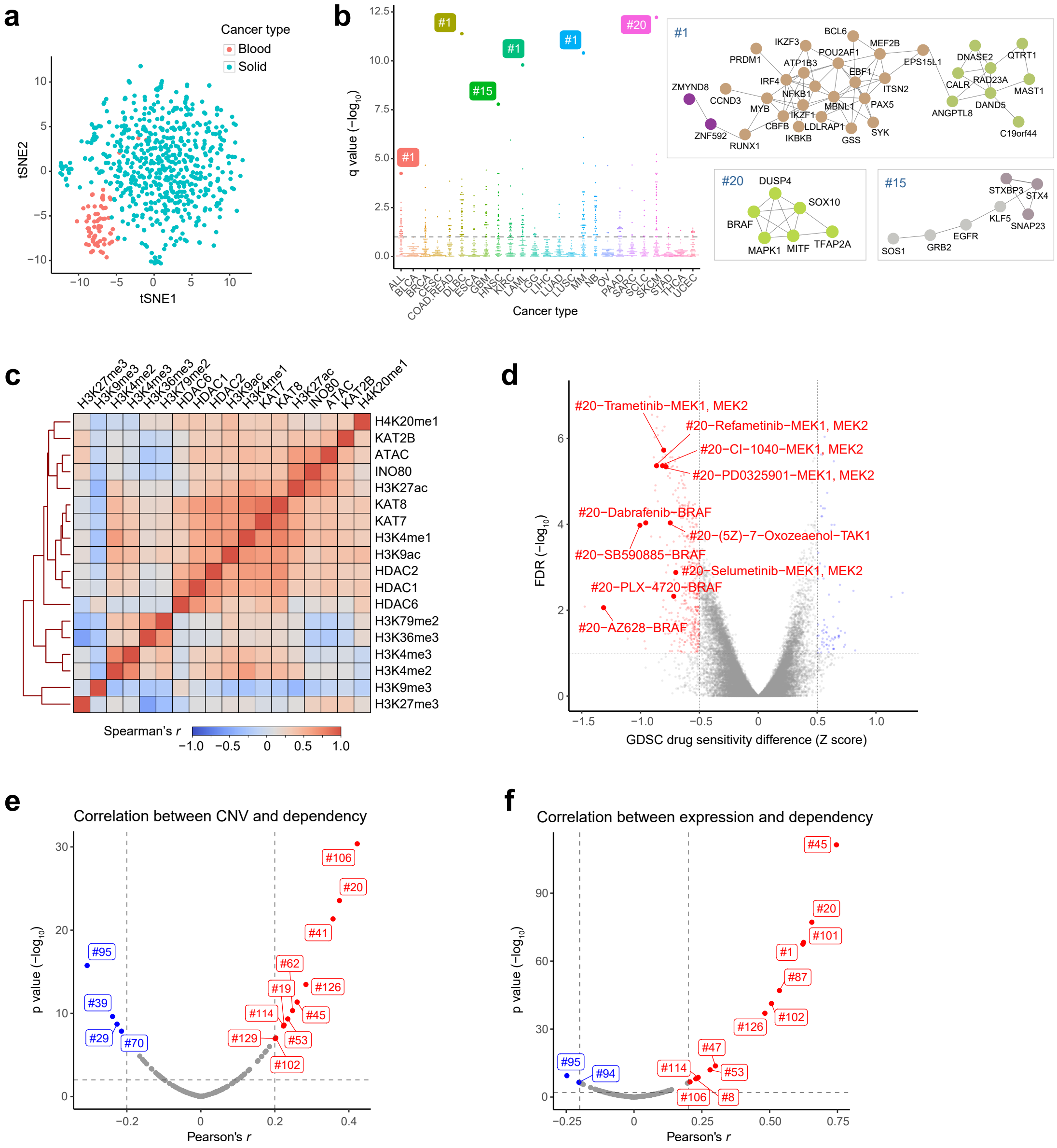

Supplementary Fig. 2: Context-specific genetic dependency of modules, related to Figure 1.

a tSNE plot showing the unsupervised clustering of 1,086 pan-cancer cell lines based on the genetic dependency of 145 small modules. Blood cancer and solid tumor cell lines are in red and green colors, respectively.

b Dot plot showing the cancer type-specific dependency of genetic modules. n=1086 cell lines.

c Heatmap showing the genomic co-localization of INO80 complex and open chromatin, histone acetylation and binding of various histone acetyltransferases.

d Volcano plot showing the correlation between the genetic dependency of functional modules and drug sensitivity (GDSC). The cell lines with high dependency of module #20 (BRAF, MAPK1, MITF, etc.) are also more sensitive to drugs that target BRAF/MEK1/MEK2 pathway.

e Volcano plot showing the correlation between gene-level copy number variation (CNV) data and dependency scores of gene members in each module across pan-cancer cell lines. 686 cell lines from cancer types containing at least ten cell lines were analyzed.

f Volcano plot showing the correlation between expression levels and dependency scores of gene members in each module across pan-cancer cell lines. 686 cell lines from cancer types containing at least ten cell lines were analyzed.

For all experiments, p values were determined by unpaired two-tailed Student's *t*-test. Source data are provided in Supplementary Data 13, Supplementary Data 14 and as a Source Data file.

Supplementary Fig. 3

a

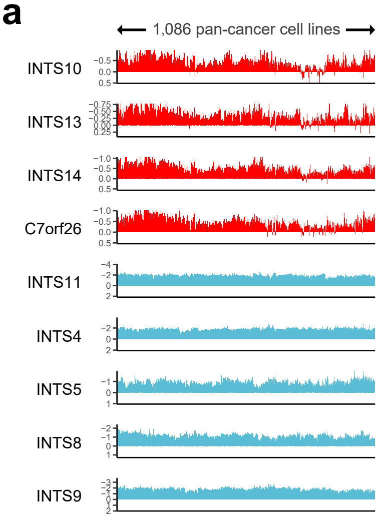

b

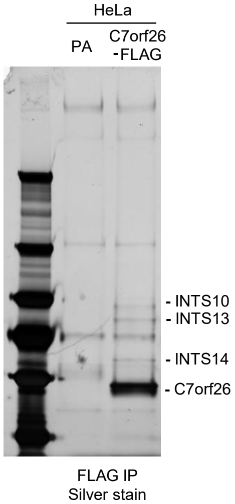

c

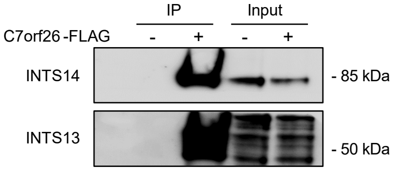

d

Identified Proteins	Molecular Weight	Unique peptide number	
		C7orf26-FLAG	Parental
**C7orf26 (bait)**	**50 kDa**	**18**	**0**
**INTS13**	**80 kDa**	**30**	**0**
**INTS10**	**82 kDa**	**28**	**0**
**INTS14**	**57 kDa**	**19**	**0**
INTS5	108 kDa	0	0
INTS8	113 kDa	0	0
INTS4	108 kDa	0	0
INTS9	74 kDa	0	0
INTS11	68 kDa	0	0

e

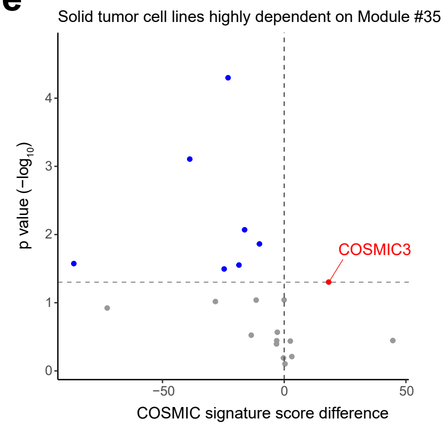

Supplementary Fig. 3: DCN reveals novel complex-level protein interactions of integrators, related to Figure 2.

a Dependency landscape of the integrator complex members across 1,086 pan-cancer cell lines (x-axis). Y-axis indicates gene effect scores. A smaller score indicates that a gene is more likely to be dependent in a given cell line.

b Silver stain showing immunoprecipitated proteins using anti-FLAG antibody in either parental and C7orf26-FLAG-expressing HeLa cells. The experiments were repeated twice independently with similar results.

c Western blot showing the co-immunoprecipitation of INTS13, INTS14 with FLAG-tagged C7orf26 in HeLa cells. The experiments were repeated twice independently with similar results.

d Mass spectrometry of immunoprecipitated proteins using anti-FLAG antibody in either parental and C7orf26-FLAG-expressing HeLa cells.

e Volcano plot showing solid tumor cell lines that highly dependent on module #35 (C7orf26, INTS10/13/14) have strong COMSIC3 signature. 616 cell lines from solid tumors containing at least ten cell lines were analyzed. P values were determined by unpaired two-tailed Student's *t*-test. For statistical analyses of COSMIC signature score, p (COSMIC3) = 0.0498.

Source data are provided as a Source Data file.

Supplementary Fig. 4

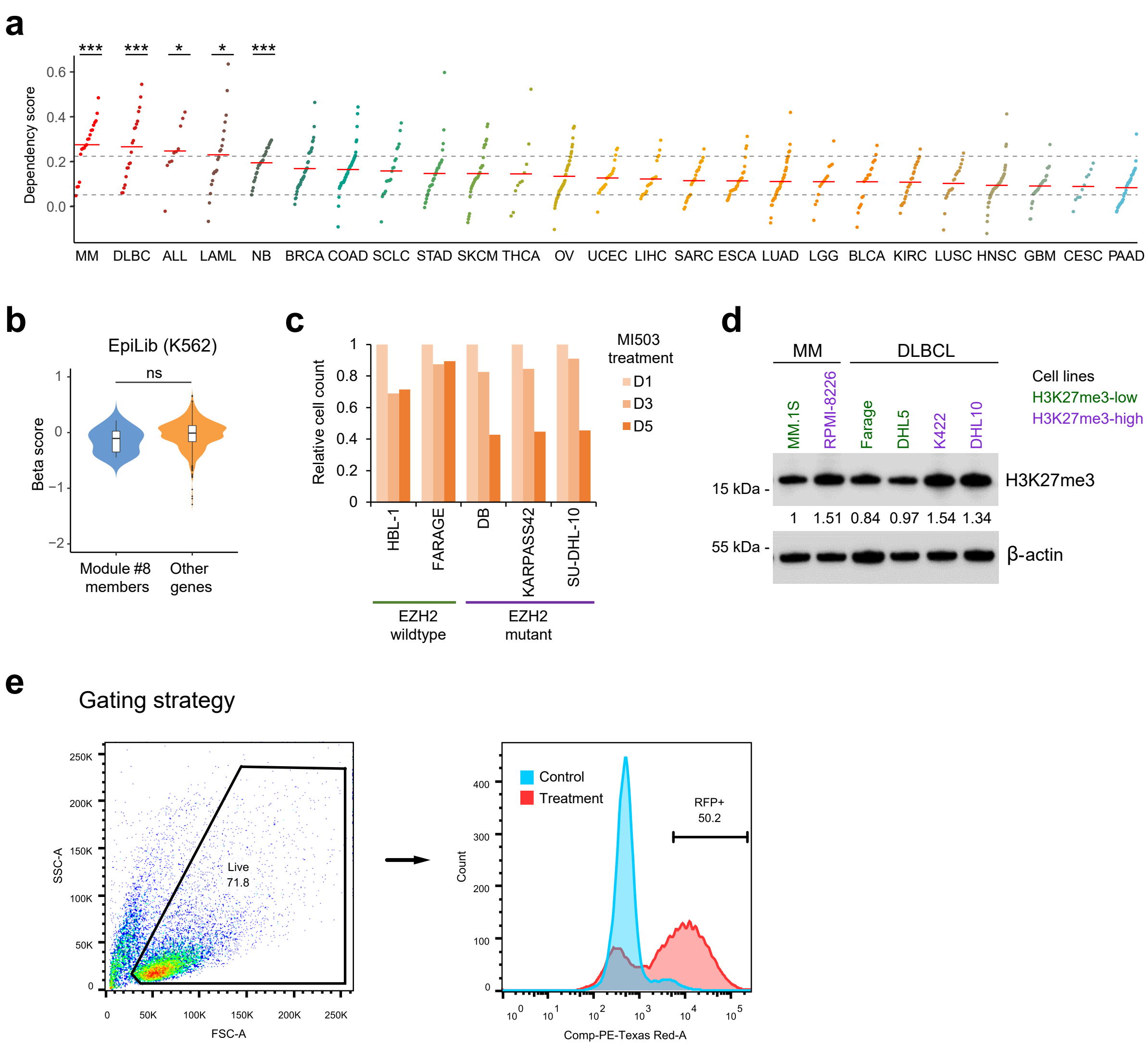

Supplementary Fig. 4: CRISPR-Cas9 essentiality screens reveal preferential dependency of MLL-MEN1 in EZH2-mutated DLBCL, related to Figure 3.

a Dot plot showing the dependency landscape of module #8 in cell lines across cancer types. n=20, 20, 10, 20 and 27 cell lines for MM, DLBC, ALL, LAML and NB, respectively. Mean dependency scores are denoted by red bars. For statistical analyses of dependency score, p (MM) = 3.40e-5; p (DLBC) = 2.68e-4; p (ALL) = 8.90e-4; p (LAML) = 0.0232; p (NB) = 0.0236.

b Violin plot showing that the difference in genetic dependency between gene members of module #8 (MLL-MEN1, PRC2 complex, etc., n=12 genes) and other genes (n=553 genes) in our EpiLib screen in EZH2 wildtype leukemia cell line K562 is not significant. The center line in the embedded boxplots represents the median, the box limits are the 25th and 75th percentiles, and the whiskers are the minimum to maximum values.

c Relative percentage of viable cells of DLBCL cell lines treated with 800 nM of MEN1 inhibitor MI503 for five days, normalized to the DMSO-treated controls.

d Western blot showing the H3K27me3 levels in MM cell lines and DLBCL cell lines, using β-actin as control. H3K27me3-low and H3K27me3-high cell lines are labeled in blue and orange, respectively. Relative abundance of H3K27me3 after control normalization of each cell line compared to that of MM.1S cell line is denoted. The experiments were repeated twice independently with similar results.

e A representative gating strategy of the flow cytometric analysis of RFP marker.

For all experiments, p values were determined by unpaired two-tailed Student's *t*-test. *, $p < 0.05$; ***, $p < 0.001$; ns, not significant. Source data are provided as a Source Data file.

Supplementary Fig. 5

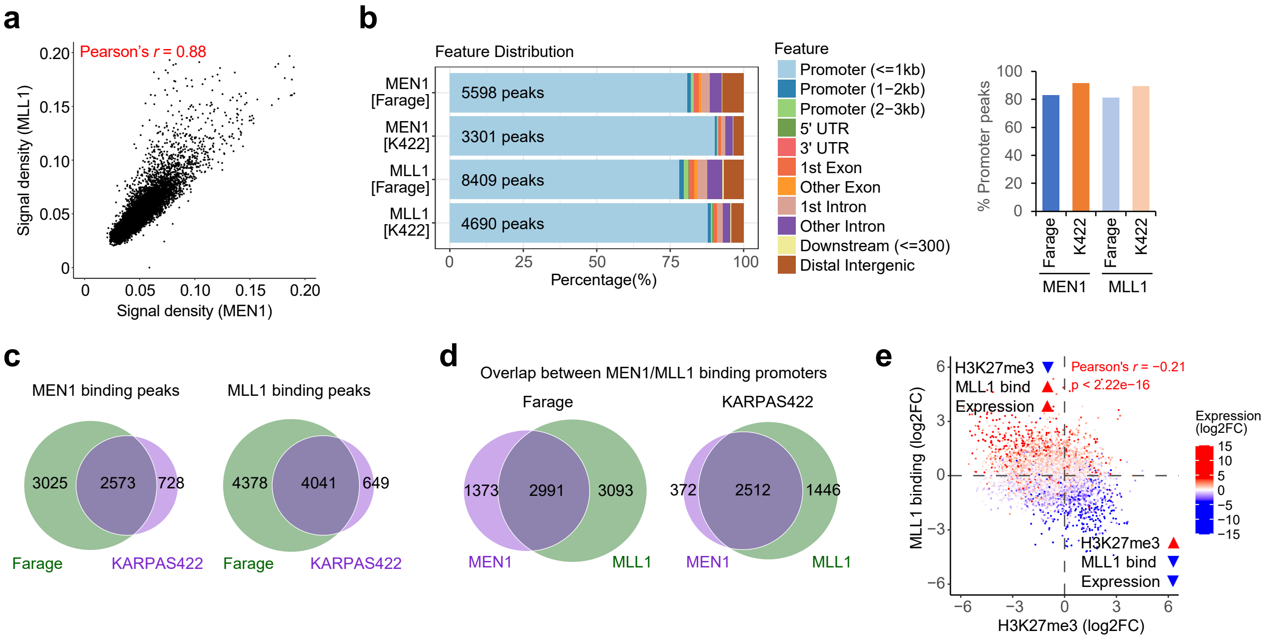

Supplementary Fig. 5: Distribution of H3K27me3 and MEN1/MLL1 binding in DLBCL cell lines Farage and KARPAS422, related to Figure 4.

a Density plot showing the correlation between genome-wide bindings of MEN1 and MLL1 in KARPAS422 (bin size = 100 kb).

b Annotation of MEN1 and MLL1 binding peaks in Farage and KARPAS422 cells (left). Bar plot showing the percentage of promoters among MLL1 and MEN1 peaks in Farage and KARPAS422 cells (right).

c Venn diagrams showing the overlap of MEN1 (left) or MLL1 (right) binding peaks between KARPAS422 (purple) and Farage (green) cells.

d Venn diagrams showing the overlap between MEN1 (purple) and MLL1 (green) binding promoters in Farage (left) and KARPAS422 (right) cells.

e Scatter plot showing the correlation between the difference in H3K27me3 abundance (x-axis), MLL1 binding (y-axis) at gene promoter regions and corresponding gene expression changes (log2 of fold change, colored in blue and red for down- and upregulation, respectively) between KARPAS422 and Farage (KARPAS422 vs. Farage). P value was determined by one-way ANOVA.

Source data are provided in Supplementary Data 10 and as a Source Data file.

Supplementary Fig. 6

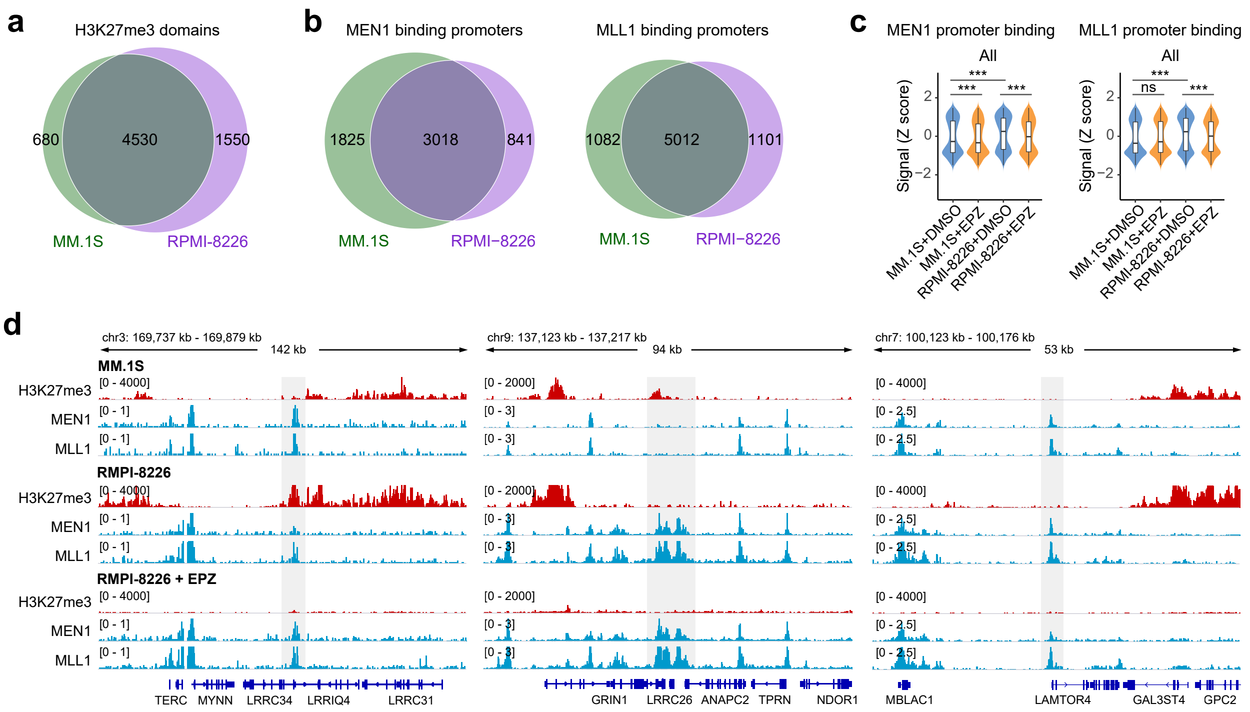

Supplementary Fig. 6: Distribution of H3K27me3 and MEN1/MLL1 binding in MM cell lines MM.1S and RPMI-8226, related to Figure 4.

a Venn diagram showing the genomic partition (Mb) of H3K27me3 large domains (> 100 kb) shared between H3K27me3-high MM cell line RPMI-8226 (purple) and H3K27me3-low MM cell line MM.1S (green).

b Venn diagrams showing the MEN1 (left) or MLL1 (right) binding promoters shared between RPMI-8226 (purple) and MM.1S (green) cells.

c Violin plots showing the normalized signal abundance (Z score) of MEN1 (left) and MLL1 binding (right) at all MEN1/MLL1-bound promoters (n=6638 and 9012 for all MEN1/MLL1-bound promoters, respectively) in MM.1S and RPMI-8226 cells (all). EPZ, EZH2 inhibitor EPZ-6438. The center line in the embedded boxplots represents the median, the box limits are the 25th and 75th percentiles, and the whiskers are the minimum to maximum values. P values were determined by unpaired two-tailed Student's *t*-test. ***, $p < 0.001$; ns, not significant. For statistical analyses of MEN1 in promoters, p (MM.1S+DMSO vs. RPMI-8226+DMSO) < 2.22e-16; p (MM.1S: DMSO vs. EPZ) = 0.0007415; p (RPMI-8226: DMSO vs. EPZ) < 2.22e-16. For statistical analyses of MLL1 in promoters, p (MM.1S+DMSO vs. RPMI-8226+DMSO) < 2.22e-16; p (MM.1S: DMSO vs. EPZ) = 0.09944; p (RPMI-8226: DMSO vs. EPZ) < 5.17e-15.

d Integrative Genomics Viewer snapshot showing the landscape of H3K27me3, MEN1 and MLL1 binding at the LRRC34 (left), LRRC26 (middle) and LAMTOR4 (right) gene loci in MM cell lines. EPZ, EZH2 inhibitor EPZ-6438.

Source data are provided as a Source Data file.

Supplementary Fig. 7

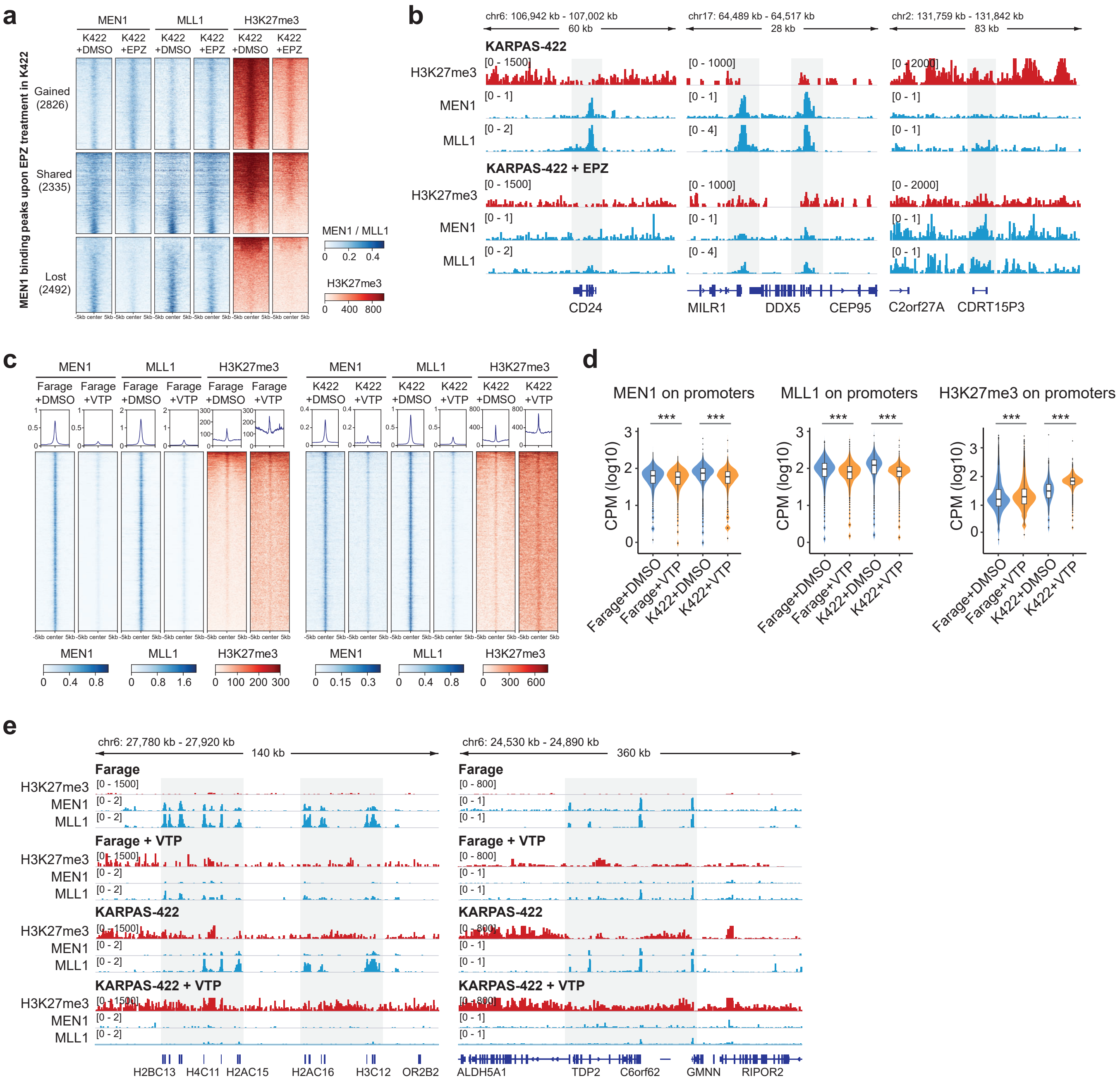

Supplementary Fig. 7: EZH2i/MEN1i treatment redistributes MEN1 binding and H3K27me3 signals, related to Figure 4.

a Heatmaps showing the corresponding levels of MEN1 and MLL1 binding, and H3K27me3 around gained (top), shared (middle) and lost (bottom) MEN1 binding peaks upon EPZ-6438 treatment in KARPAS422. Each row represents one MEN1 binding peak and shows CUT&Tag signal intensity (CPM) within 5 kb range on each side of the peak center.

b Integrative Genomics Viewer snapshot showing the landscape of H3K27me3, MEN1 and MLL1 binding at the CD24 (left), DDX5 (middle) and CDRT15P3 (right) gene loci in KARPAS422 cell treated with EPZ-6438 or DMSO.

c Heatmaps showing the corresponding levels of MEN1 and MLL1 binding, and H3K27me3 around MEN1 binding peaks upon VTP-50469 treatment in Farage (left) and KARPAS422 cells (right). Each row represents one MEN1 binding peak and shows CUT&Tag signal intensity (CPM) within 5 kb range on each side of the peak center.

d Violin plots showing the normalized signal abundance (CPM) of MEN1 (left) and MLL1 binding (middle), and H3K27me3 (right) at all MEN1-bound promoters (within 5 kb range on each side of the promoter center, n= 3012 and 1979 for all MEN1-bound promoters in Farage and KARPAS422 cells, respectively) in Farage and KARPAS422 cells. VTP, MEN1 inhibitor VTP-50469. The center line in the embedded boxplots represents the median, the box limits are the 25th and 75th percentiles, and the whiskers are the minimum to maximum values. P values were determined by unpaired two-tailed Student's *t*-test. ***, $p < 0.001$. For statistical analyses of MEN1 on promoters, p (Farage: DMSO vs. VTP) = 5.71e-4; p (KARPAS422: DMSO vs. VTP) = 7.23e-7. For statistical analyses of MLL1 on promoters, p (Farage: DMSO vs. VTP) = 1.01e-8; p (KARPAS422: DMSO vs. VTP) = 7.86e-16. For statistical analyses of H3K27me3 on promoters, p (Farage: DMSO vs. VTP) = 2.17e-5; p (KARPAS422: DMSO vs. VTP) < 2.22e-16.

e Integrative Genomics Viewer snapshot showing the landscape of H3K27me3, MEN1 and MLL1 binding at the H4C11 (left), TDP2 (right) gene loci in Farage and KARPAS422 cells treated with VTP-50469 or DMSO.

Source data are provided as a Source Data file.

Supplementary Fig. 8

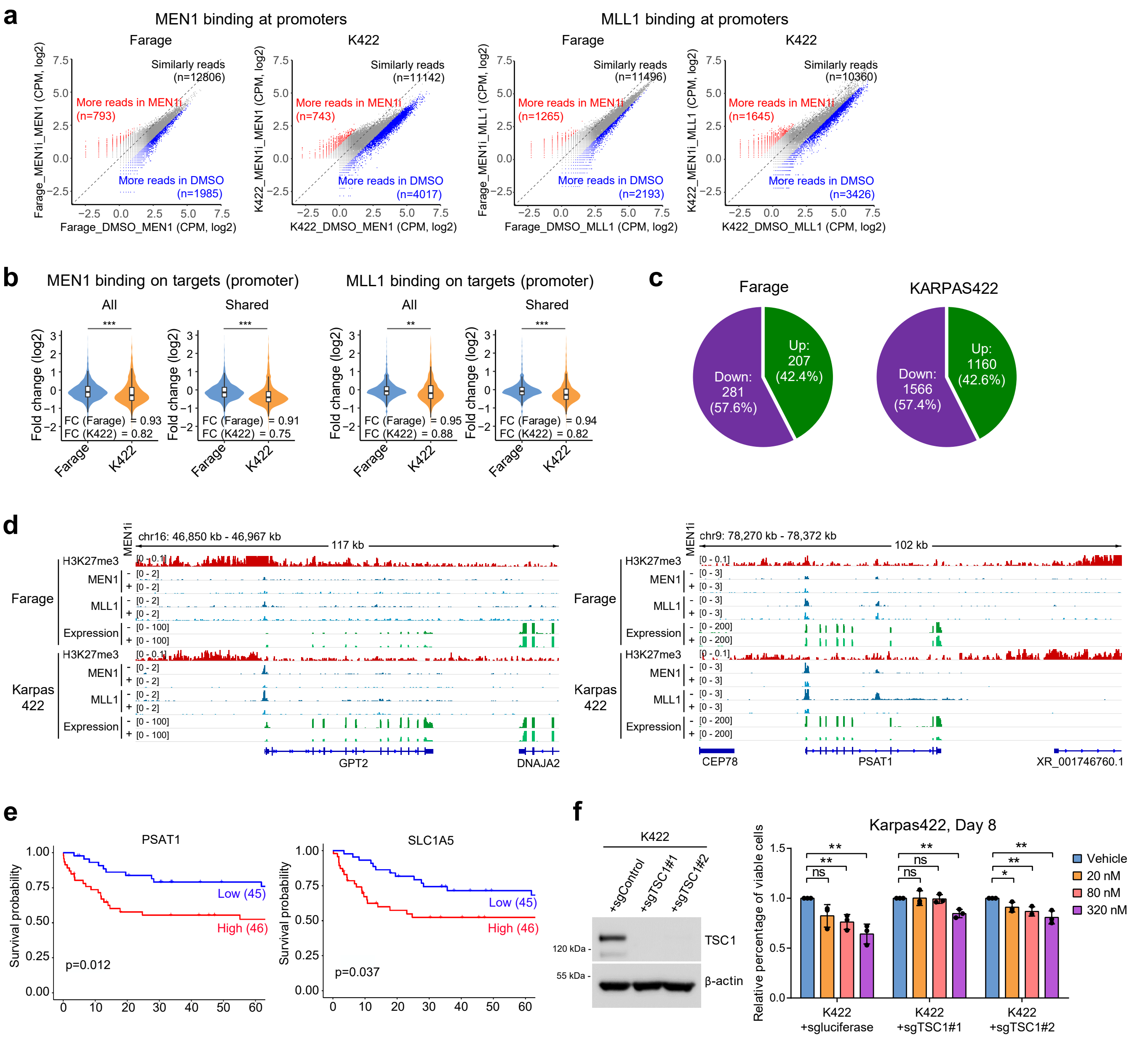

Supplementary Fig. 8: Differential sensitivity to MEN1 inhibitor between Farage and KARPAS422 cells, related to Figure 5.

a Dot plot showing the signal abundance changes of MEN1 (left) and MLL1 (right) at gene promoters in Farage and KARPAS422 cells upon MEN1 inhibitor treatment (1 μM of MI-503, treated for 7 days).

b Violin plots showing the fold change in MEN1 and MLL1 binding upon MEN1 inhibitor treatment for all MEN1/MLL1-bound promoters in Farage and KARPAS422 cells (all, n=5075 and 6813 for all MEN1/MLL1-bound promoters, respectively) or only those promoters shared between Farage and KARPAS422 cells (shared, n=2305 and 3562 for shared MEN1/MLL1-bound promoters, respectively). The center line in the embedded boxplots represents the median, the box limits are the 25th and 75th percentiles, and the whiskers are the minimum to maximum values. P values were determined by unpaired two-tailed Student's *t*-test. **, $p < 0.01$; ***, $p < 0.001$. For statistical analyses of MEN1 binding, p (All) = 2.05e-10; p (Share) < 2.22e-16. For statistical analyses of MLL1 binding, p (All) = 0.00289; p (Share) < 2.22e-16.

c Pie charts showing the number of genes that are up-/down-regulated in Farage (left) and KARPAS422 cells upon MEN1 inhibitor treatment (FDR < 0.1).

d Integrative Genomics Viewer snapshot showing the landscape of H3K27me3, MEN1 and MLL1 binding, and gene transcription at the GPT2 (left) and PSAT1 (right) loci before and after MEN1 inhibitor treatment in Farage and KARPAS422.

e Kaplan–Meier models of progression-free survival of DLBCL patients stratified by high (top 20%) or low (bottom 20%) expression of PSAT1 or SLC1A5. Log-rank (Mantel-Cox) test was used to calculate statistical significance. n=234 analyzed patients.

f Left, Western blot showing the complete knock-out of TSC1 in KARPAS422 cells using sgRNAs. Right, relative percentage of viable cells of KARPAS422 with or without TSC1 knock-out under VTP-50469 treatment in different dosage for 8 days, normalized to the DMSO-treated controls. n=3 independent experiments. Bar plots and whiskers are mean ± s.d. P values were determined by unpaired two-tailed Student's *t*-test. *, $p < 0.05$; **, $p < 0.01$; ns, not significant. For statistical analyses of KARPAS422+sgluciferase, p (20 nM vs. Vehicle) = 0.0542; p (80 nM vs. Vehicle) = 0.00520; p (320 nM vs. Vehicle) = 0.00333. For statistical analyses of KARPAS422+sgTSC1#1, p (20 nM vs. Vehicle) = 0.968; p (80 nM vs. Vehicle) = 0.799; p (320 nM vs. Vehicle) = 0.00329. For statistical analyses of KARPAS422+sgTSC1#2, p (20 nM vs. Vehicle) = 0.0346; p (80 nM vs. Vehicle) = 0.00780; p (320 nM vs. Vehicle) = 0.00702.

Source data are provided as a Source Data file.

Supplementary Fig. 9

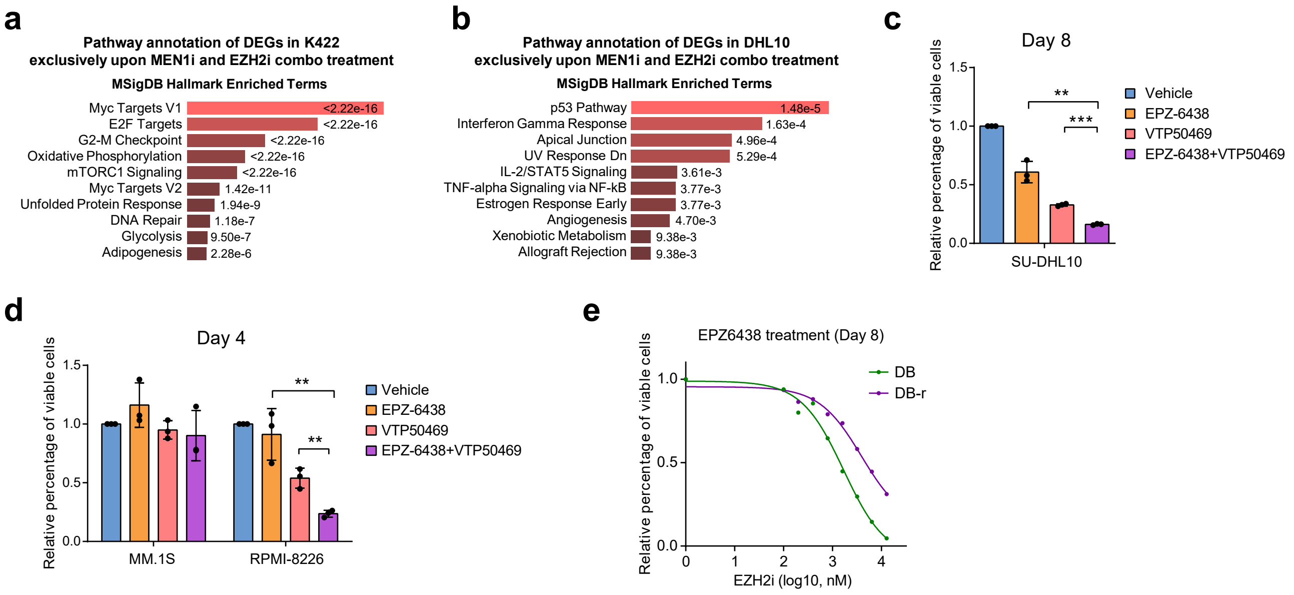

Supplementary Fig. 9: Inhibition of both MEN1 and EZH2 in EZH2-mutated DLBCL, related to Figure 6.

a Pathway annotation of exclusively differentially expressed genes in KARPAS422 upon combo treatment by both MEN1 and EZH2 inhibitors.

b Pathway annotation of exclusively up-regulated genes (left) and exclusively differentially expressed genes (right) in SuDHL10 upon combo treatment by both MEN1 and EZH2 inhibitors.

c Relative percentage of viable cells of DLBCL cell line SuDHL10 under VTP-50469 (red) or EPZ-6438 (yellow) treatment, and combo treatment by both inhibitors (purple) for 8 days, normalized to the DMSO-treated controls. n=3 independent experiments. Bar plots and whiskers are mean ± s.d. P values were determined by unpaired two-tailed Student's *t*-test. **, $p < 0.01$; ***, $p < 0.001$. For statistical analyses of relative percentage of viable cells, p (EPZ-6438 vs. EPZ-6438+VTP50469) = 0.0011; p (VTP50469 vs. EPZ-6438+VTP50469) = 1.78e-5.

d Relative percentage of viable cells of MM cell lines under VTP-50469 (red) or EPZ-6438 (yellow) treatment, and combo treatment by both inhibitors (purple) for 4 days, normalized to the DMSO-treated controls (n=3). Bar plots and whiskers are mean ± s.d. P values were determined by unpaired two-tailed Student's *t*-test. **, $p < 0.01$. For statistical analyses of relative percentage of viable cells of RPMI-8226, p (EPZ-6438 vs. EPZ-6438+VTP50469) = 0.00618; p (VTP50469 vs. EPZ-6438+VTP50469) = 0.00436.

e Dose-response curve validating the establishment of DB cells resistant to EZH2 inhibitor (EPZ6438). The experiment is not repeated.

Source data are provided as a Source Data file.

Supplementary Fig. 10

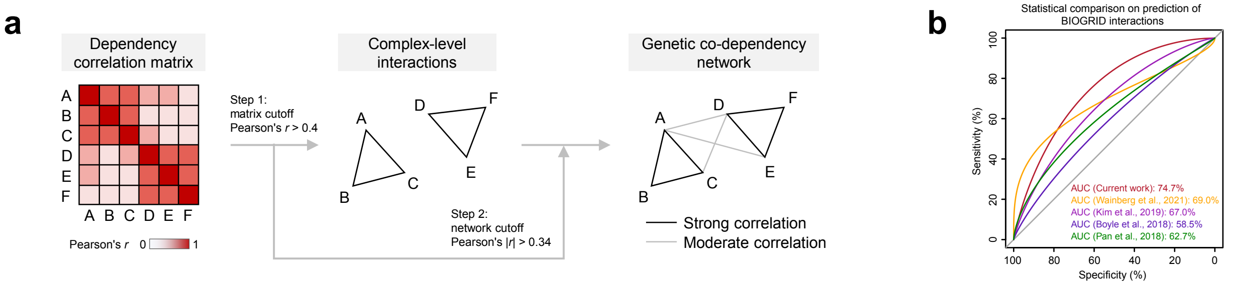

Supplementary Fig. 10: A complex-centric strategy for identifying genetic interactions from dependency correlation network based on CRISPR-Cas9-based screening datasets.

a A scheme for the complex-centric strategy using two-step cutoff for correlation coefficient used in the current work. Black and grey lines denote strong and moderate correlations between genetic dependencies, respectively.

b Statistical comparison on prediction of BIOGRID interactions between the current work and recent studies. AUC, area under curve.
