# Supplementary material for: Context-defined cancer co-dependency mapping identifies a functional interplay between PRC2 and MLL-MEN1 complex in lymphoma
Source: Nat Commun. 2023 Jul 17;14:4259. doi: 10.1038/s41467-023-39990-5 (PMC10352330; doi:10.1038/s41467-023-39990-5)
Supplement: Supplementary file 3 — Description of Additional Supplementary Files [file 41467_2023_39990_MOESM3_ESM.pdf]

Description of Additional Supplementary Files

Supplementary Data 1. Cross comparison of genetic interactions with strong essentiality correlation identified in the current study and BioGRID database.

Supplementary Data 2. Dependency correlation matrix for 590 genes in co-essentiality network, related to Supplementary Figure 1c.

Supplementary Data 3. List of genes in dependency correlation network modules, related to Supplementary Figure 1d.

Supplementary Data 4. Differential essentiality of dependency correlation network modules based on cancer types, related to Figure 1b.

Supplementary Data 5. Differential essentiality of dependency correlation network modules based on COSMIC signatures in solid tumor cell lines, related to Figure 1c.

Supplementary Data 6. Differential essentiality of dependency correlation network modules based on chromatin modifications in blood cancer cell lines, related to Figure 1d.

Supplementary Data 7. Differential essentiality of dependency correlation network modules based on GSEA hallmark in cancer cell lines, related to Figure 1b.

Supplementary Data 8. Correlation between dependency of DCN modules and GDSC drug sensitivity in cancer cell lines, related to Figure 1e.

Supplementary Data 9. Mass spectrometry report of peptides from immunoprecipitation by anti-FLAG in parental and C7orf26-FLAG HeLa cells, related to Supplementary Figure 3d.

Supplementary Data 10. Annotation of MLL1 and MEN1 binding peaks in Farage and KARPAS422, related to Supplementary Figure 5b.

Supplementary Data 11. Differentially expressed genes (fold change>1.5, q<0.1) upon MI-503 treatment in Farage, SUDHL5, KARPAS422 and DB cells, related to Figure 5.

Supplementary Data 12. Comparison of the current work with recent studies of building pan-cancer genetic co-dependency networks from CRISPR-Cas9-based screening datasets.

Supplementary Data 13. Correlation between genetic dependency and copy number variation of gene members in each DCN module, related to Supplementary Figure 2e.

Supplementary Data 14. Correlation between genetic dependency and expression levels of gene members in each DCN module, related to Supplementary Figure 2f.

Supplementary Data 15. Information of sgRNAs used in CRISPR-Cas9 gene knockout.
