# Supplementary material for: Context-defined cancer co-dependency mapping identifies a functional interplay between PRC2 and MLL-MEN1 complex in lymphoma
Source: Nat Commun. 2023 Jul 17;14:4259. doi: 10.1038/s41467-023-39990-5 (PMC10352330; doi:10.1038/s41467-023-39990-5)
Supplement: Supplementary file 5 — Reporting Summary [file 41467_2023_39990_MOESM5_ESM.pdf]

nature portfolio

Corresponding author(s): Chao Lu

Last updated by author(s): Jun 15, 2023

Reporting Summary

Nature Portfolio wishes to improve the reproducibility of the work that we publish. This form provides structure for consistency and transparency in reporting. For further information on Nature Portfolio policies, see our Editorial Policies and the Editorial Policy Checklist.

Statistics

For all statistical analyses, confirm that the following items are present in the figure legend, table legend, main text, or Methods section.

n/a	Confirmed	
☐	☒	The exact sample size (*n*) for each experimental group/condition, given as a discrete number and unit of measurement
☐	☒	A statement on whether measurements were taken from distinct samples or whether the same sample was measured repeatedly
☐	☒	The statistical test(s) used AND whether they are one- or two-sided<br>*Only common tests should be described solely by name; describe more complex techniques in the Methods section.*
☐	☒	A description of all covariates tested
☒	☐	A description of any assumptions or corrections, such as tests of normality and adjustment for multiple comparisons
☐	☒	A full description of the statistical parameters including central tendency (e.g. means) or other basic estimates (e.g. regression coefficient) AND variation (e.g. standard deviation) or associated estimates of uncertainty (e.g. confidence intervals)
☐	☒	For null hypothesis testing, the test statistic (e.g. *F*, *t*, *r*) with confidence intervals, effect sizes, degrees of freedom and *P* value noted<br>*Give P values as exact values whenever suitable.*
☒	☐	For Bayesian analysis, information on the choice of priors and Markov chain Monte Carlo settings
☒	☐	For hierarchical and complex designs, identification of the appropriate level for tests and full reporting of outcomes
☐	☒	Estimates of effect sizes (e.g. Cohen's *d*, Pearson's *r*), indicating how they were calculated

Our web collection on statistics for biologists contains articles on many of the points above.

Software and code

Policy information about availability of computer code

Data collection	BD FACSverse cytometer driven by FACSuite v1 was used to collect flow cytometry data.<br>ChemiDoc Touch Imaging System was used to collect western blot data.
Data analysis	R packages pROC (v1.18.2), pheatmap (Pretty Heatmaps v1.0.10), ChIPseeker, DESeq2 (v1.28.0) and ggplot2 (v3.2.1)<br>HISAT2 (v2.1.0)<br>featureCounts (v1.6.1)<br>GSEA software (v4.1.0)<br>Picard (v2.23.1)<br>SICER (v2)<br>bedtools (v2.27.1)<br>deepTools (v 3.3.2)<br>FlowJo (V10)<br>GraphPad Prism (v9)

For manuscripts utilizing custom algorithms or software that are central to the research but not yet described in published literature, software must be made available to editors and reviewers. We strongly encourage code deposition in a community repository (e.g. GitHub). See the Nature Portfolio guidelines for submitting code & software for further information.

Data

Policy information about availability of data

All manuscripts must include a data availability statement. This statement should provide the following information, where applicable:

- Accession codes, unique identifiers, or web links for publicly available datasets
- A description of any restrictions on data availability
- For clinical datasets or third party data, please ensure that the statement adheres to our policy

The publicly available gene effect dataset of CRISPR-Cas9 essentiality screens in 1,086 pan-cancer cell lines (gene effect scores derived from CRISPR knockout screens published by Broad's Achilles and Sanger's SCORE projects, release 2022q2) used in this study is available in the Cancer Dependency Map portal (DepMap) [https://depmap.org/portal]. The publicly available human core complexes data used in this study are available in the CORUM database [https://mips.helmholtz-muenchen.de/corum]. The publicly available genetic or physical interaction data used in this study are available in the BioGRID database [https://downloads.thebiogrid.org/BioGRID]. The pan-cancer genetic co-dependency networks built from CRISPR-Cas9-based screening datasets in recent studies are publicly available in their supplementary materials and figshare [https://figshare.com/s/35a82ed1e48d0ec4e9e4]. The publicly available genome-wide binding data of INO80 complex and various chromatin features used in this study are available in the NCBI GEO database under accession code GSE97411 [https://www.ncbi.nlm.nih.gov/geo/query/acc.cgi?acc=GSE97411]. The publicly available survival information and gene expression data of a DLBCL patient cohort are available in the supplementary materials of a previous report and GDC Data Portal [https://gdc.cancer.gov/about-data/publications/DLBCL-2018]. The complete output of analysis using Deplink is provided in figshare [https://doi.org/10.6084/m9.figshare.21708425.v1] and can be queried via a searchable database (http://www.chaolulab-database.com). The raw sequencing data generated in this study (CUT&Tag, RNA-seq and CRISPR-Cas9 genetic screening) are available in the NCBI GEO database under accession code GSE183487 [https://www.ncbi.nlm.nih.gov/geo/query/acc.cgi?acc=GSE183487]. The mass spectrometry raw data generated in this study are available in the ProteomeXchange member PRIDE database under accession code PXD033140 [https://dx.doi.org/10.6019/PXD033140]. Source data are provided with this paper.

Research involving human participants, their data, or biological material

Policy information about studies with human participants or human data. See also policy information about sex, gender (identity/presentation), and sexual orientation and race, ethnicity and racism.

Reporting on sex and gender	N/A
Reporting on race, ethnicity, or other socially relevant groupings	N/A
Population characteristics	N/A
Recruitment	N/A
Ethics oversight	N/A

Note that full information on the approval of the study protocol must also be provided in the manuscript.

Field-specific reporting

Please select the one below that is the best fit for your research. If you are not sure, read the appropriate sections before making your selection.

[x] Life sciences [ ] Behavioural & social sciences [ ] Ecological, evolutionary & environmental sciences

For a reference copy of the document with all sections, see nature.com/documents/nr-reporting-summary-flat.pdf

Life sciences study design

All studies must disclose on these points even when the disclosure is negative.

Sample size	The gene effect dataset of CRISPR-Cas9 essentiality screens in 1,086 pan-cancer cell lines (gene effect scores derived from CRISPR knockout screens published by Broad's Achilles and Sanger's SCORE projects, release public 2022q2) was downloaded from Cancer Dependency Map portal (DepMap, https://depmap.org/portal/). The number of samples for each assay was indicated in each figure legend. The sample size was determined by using power calculation for a t-test difference between two or three independent means based on a normally distributed population with equal variance.
Data exclusions	No data was excluded from analysis.
Replication	We included two replicates for CRISPR knockouts using individual sgRNAs and performed three replicates for cell proliferation experiments. Replicated experiments were successful and support conclusions drawn in this report.
Randomization	Animals were randomly assigned to two or more groups prior to the injection of cells or drug.
Blinding	The investigators were not blinded for any of the experiments, including to the animal assignments for tumor formation studies and molecular analysis as treatment conditions were evident from the data.

Reporting for specific materials, systems and methods

We require information from authors about some types of materials, experimental systems and methods used in many studies. Here, indicate whether each material, system or method listed is relevant to your study. If you are not sure if a list item applies to your research, read the appropriate section before selecting a response.

Materials & experimental systems

n/a	Involved in the study
☐	☒ Antibodies
☐	☒ Eukaryotic cell lines
☒	☐ Palaeontology and archaeology
☐	☒ Animals and other organisms
☒	☐ Clinical data
☒	☐ Dual use research of concern
☒	☐ Plants

Methods

n/a	Involved in the study
☐	☒ ChIP-seq
☐	☒ Flow cytometry
☒	☐ MRI-based neuroimaging

Antibodies

Antibodies used

anti-MEN1 (Bethyl, Cat#A300-105A, Lot#11, 1:50 dilution for CUT&Tag, 1:2000 dilution for WB)
anti-MLL1 (Bethyl, Cat#A300-086A, Lot#6, 1:50 dilution for CUT&Tag, 1:2000 dilution for WB)
anti-H3K27me3 (Cell Signaling, Cat#9733, Clone C36B11, Lot#19, 1:100 dilution for CUT&Tag, 1:1000 dilution for WB)
anti-IgG (Cell Signaling, Cat#2729, Lot#9, 1:100 dilution for CUT&Tag)
anti-INTS13 (Bethyl, Cat#A303-575A, 1:1000 dilution for WB)
anti-INTS14 (Bethyl, Cat#A303-576A, 1:1000 dilution for WB)
anti-Phospho-S6 (Cell Signaling, Cat#4858, Clone D57.2.2E, 1:2000 dilution for WB)
anti-S6 (Cell Signaling, Cat#2317, Clone 54D2, 1:1000 dilution for WB)
anti-TSC1 (Cell Signaling, Cat#6935, Clone D43E2, 1:1000 dilution for WB)
anti-H3 (Abcam, Cat#ab1791, 1:10000 dilution for WB)
anti-Beta-actin (Abcam, Cat#ab8224, 1:2000 dilution for WB)

Validation

Antibodies were all validated by western blot using the manufacture's data associated with antibody, and their authentication data.
anti-MEN1 (https://www.thermofisher.com/antibody/product/Menin-Antibody-Polyclonal/A300-105A)
anti-MLL1 (https://www.thermofisher.com/antibody/product/MLL1-Antibody-Polyclonal/A300-086A)
anti-H3K27me3 (https://www.cellsignal.com/products/primary-antibodies/tri-methyl-histone-h3-lys27-c36b11-rabbit-mab/9733)
anti-IgG (https://www.cellsignal.com/products/primary-antibodies/normal-rabbit-igg/2729)
anti-INTS13 (https://www.thermofisher.com/antibody/product/Asunder-Antibody-Polyclonal/A303-575A)
anti-INTS14 (https://www.thermofisher.com/antibody/product/C15orf44-Antibody-Polyclonal/A303-576A-T)
anti-Phospho-S6 (https://www.cellsignal.com/products/primary-antibodies/phospho-s6-ribosomal-protein-ser235-236-d57-2-2e-xp-rabbit-mab/4858)
anti-S6 (https://www.cellsignal.com/products/primary-antibodies/s6-ribosomal-protein-54d2-mouse-mab/2317)
anti-TSC1 (https://www.cellsignal.com/products/primary-antibodies/hamartin-tsc1-d43e2-rabbit-mab/6935)
anti-H3 (https://www.abcam.com/products/primary-antibodies/histone-h3-antibody-nuclear-marker-and-chip-grade-ab1791.html)
anti-Beta-actin (https://www.abcam.com/products/primary-antibodies/beta-actin-antibody-mabcam-8224-loading-control-ab8224.html)

Eukaryotic cell lines

Policy information about cell lines and Sex and Gender in Research

Cell line source(s)

Human DLBCL cell lines (KARPAS-422, DB, SU-DHL-10, SU-DHL-4, Farage, HBL-1 and SU-DHL-5): a gift from Jennifer E. Amengual (KARPAS-422: Sigma-Aldrich, #06101702; DB: ATCC, #CRL-2289; SU-DHL-10: ATCC, #CRL-2963; SU-DHL-4: ATCC, #CRL-2957; Farage: ATCC, #CRL-2630; HBL-1: ABM, #T8204; SU-DHL-5: ATCC, #CRL-2958)
Human MM cell line MM.1S: a gift from Selina Chen-Kiang (ATCC, #CRL-2974)
Human MM cell line RPMI-8226: a gift from High-Throughput Screening Facility at the JP Sulzberger Columbia Genome Center (ATCC, #CCL-155)
Human leukaemia cell line K562: ATCC (#CCL-243)
Human kidney epithelial cell line HEK293T: ATCC (#CRL-11268)
Human CESC cell line HeLa: a gift from Alberto Ciccia (ATCC, #CRM-CCL-2)

Authentication

None of the cell lines used were authenticated.

Mycoplasma contamination

We confrim all cell lines were tested negative for mycoplasma contamination.

Commonly misidentified lines
(See ICLAC register)

None

Animals and other research organisms

Policy information about studies involving animals; ARRIVE guidelines recommended for reporting animal research, and Sex and Gender in Research

Laboratory animals	6-8 weeks old male athymic nu/nu mice and 6-week-old female NOD-scid IL2Rgammanull (NSG) mice were purchased from The Jackson Laboratory. Number of mice used per experiments are described in manuscript. All mice were housed under specific-pathogen free (SPF) condition under controlled temperature (20-26°C) and humidity (40-70%) with 12h/12h light/dark cycle, followed the guideline of Columbia University animal facility.
Wild animals	No wild animals were used in the study.
Reporting on sex	Both male and female mice were used in the study.
Field-collected samples	No field-collected samples were used in the study.
Ethics oversight	Institutional Animal Care and Use Committee (IACUC) at Columbia University.

Note that full information on the approval of the study protocol must also be provided in the manuscript.

Plants

Seed stocks	N/A
Novel plant genotypes	N/A
Authentication	N/A

ChIP-seq

Data deposition

- [x] Confirm that both raw and final processed data have been deposited in a public database such as GEO.
- [x] Confirm that you have deposited or provided access to graph files (e.g. BED files) for the called peaks.

Data access links<br>*May remain private before publication.*	GEO accession GSE183487: https://www.ncbi.nlm.nih.gov/geo/query/acc.cgi?acc=GSE183487
Files in database submission	Farage_IgG.bw<br>Farage_H3K27me3.bw<br>Farage_MEN1.bw<br>Farage_MLL1.bw<br>Farage_DMSO_MEN1.bw<br>Farage_DMSO_MLL1.bw<br>Farage_MI503_MEN1.bw<br>Farage_MI503_MLL1.bw<br>K422_IgG.bw<br>K422_H3K27me3.bw<br>K422_MEN1.bw<br>K422_MLL1.bw<br>K422_DMSO_MEN1.bw<br>K422_DMSO_MLL1.bw<br>K422_MI503_MEN1.bw<br>K422_MI503_MLL1.bw<br>K422_ctr_H3K27me3_spikein_Rx.bw<br>K422_ctr_MEN1.bw<br>K422_ctr_MLL1.bw<br>K422_EPZ6438_72hr_H3K27me3_spikein_Rx.bw<br>K422_EPZ6438_72hr_MEN1.bw<br>K422_EPZ6438_72hr_MLL1.bw<br>K422_VTP50469_H3K27me3_spikein_Rx.bw<br>K422_VTP50469_MEN1.bw<br>K422_VTP50469_MLL1.bw<br>Farage_ctr_H3K27me3_spikein_Rx.bw<br>Farage_VTP50469_H3K27me3_spikein_Rx.bw<br>Farage_VTP50469_MEN1.bw<br>Farage_VTP50469_MLL1.bw<br>MM.1S_EPZ6438_72hr_H3K27me3_spikein_Rx.bw<br>MM.1S_EPZ6438_72hr_MEN1.bw

MM.1S_EPZ6438_72hr_MLL1.bw
MM.1S_DMSO_H3K27me3_spikein_Rx.bw
MM.1S_DMSO_MEN1.bw
MM.1S_DMSO_MLL1.bw
RPMI_8226_EPZ6438_72hr_H3K27me3_spikein_Rx.bw
RPMI_8226_EPZ6438_72hr_MEN1.bw
RPMI_8226_EPZ6438_72hr_MLL1.bw
RPMI_8226_DMSO_H3K27me3_spikein_Rx.bw
RPMI_8226_DMSO_MEN1.bw
RPMI_8226_DMSO_MLL1.bw

Genome browser session (e.g. UCSC)

The CUT&Tag bigwig files deposited in the GEO database can be loaded and visualized in IGV.

Methodology

Replicates

No replicates involved in CUT&Tag experiments

Sequencing depth

sample,#total_reads,#uniquely_mapped_reads,reads_type
Farage_IgG,5804309,4956874,paired-end
Farage_H3K27me3,32672585,29934865,paired-end
Farage_MEN1,4489841,3989119,paired-end
Farage_MLL1,14218656,12710850,paired-end
Farage_DMSO_MEN1,3852421,3346778,paired-end
Farage_DMSO_MLL1,3450789,3021454,paired-end
Farage_MI503_MEN1,8760556,7430313,paired-end
Farage_MI503_MLL1,6416926,5466389,paired-end
K422_IgG,10772373,9298082,paired-end
K422_H3K27me3,19926333,17895053,paired-end
K422_MEN1,21103466,18348669,paired-end
K422_MLL1,7662568,6709286,paired-end
K422_DMSO_MEN1,19189690,16731545,paired-end
K422_DMSO_MLL1,14374105,12604434,paired-end
K422_MI503_MEN1,14279132,12436010,paired-end
K422_MI503_MLL1,12344591,10727891,paired-end
K422_ctr_H3K27me3_spikein_Rx,29713420,25801163,paired-end
K422_ctr_MEN1,19068151,16483434,paired-end
K422_ctr_MLL1,13388759,11440396,paired-end
K422_EPZ6438_72hr_H3K27me3_spikein_Rx,8932703,7650405,paired-end
K422_EPZ6438_72hr_MEN1,25707702,19665864,paired-end
K422_EPZ6438_72hr_MLL1,22543683,19267172,paired-end
K422_VTP50469_H3K27me3_spikein_Rx,19569576,17036389,paired-end
K422_VTP50469_MEN1,29502854,13767156,paired-end
K422_VTP50469_MLL1,19041252,15077066,paired-end
Farage_ctr_H3K27me3_spikein_Rx,16304127,14032839,paired-end
Farage_VTP50469_H3K27me3_spikein_Rx,14306282,11410351,paired-end
Farage_VTP50469_MEN1,20546108,14137963,paired-end
Farage_VTP50469_MLL1,15885653,9946498,paired-end
MM.1S_EPZ6438_72hr_H3K27me3_spikein_Rx,19228262,15215099,paired-end
MM.1S_EPZ6438_72hr_MEN1,16925365,10267494,paired-end
MM.1S_EPZ6438_72hr_MLL1,18879602,14017862,paired-end
MM.1S_DMSO_H3K27me3_spikein_Rx,15093732,12999078,paired-end
MM.1S_DMSO_MEN1,18323897,11869444,paired-end
MM.1S_DMSO_MLL1,15502134,9817649,paired-end
RPMI_8226_EPZ6438_72hr_H3K27me3_spikein_Rx,13389013,10777092,paired-end
RPMI_8226_EPZ6438_72hr_MEN1,16696690,12280893,paired-end
RPMI_8226_EPZ6438_72hr_MLL1,15356445,11485034,paired-end
RPMI_8226_DMSO_H3K27me3_spikein_Rx,16801925,14008328,paired-end
RPMI_8226_DMSO_MEN1,16070368,11809562,paired-end
RPMI_8226_DMSO_MLL1,13795233,10365500,paired-end

Antibodies

anti-MEN1 (Bethyl, #A300-105A), anti-MLL1 (Bethyl, #A300-086A), anti-H3K27me3 (Cell Signaling, #9733), anti-IgG (Cell Signaling, #2729)

Peak calling parameters

Broad H3K27me3 peaks were called using SICER2 (parameters: -w 10000 -g 30000 -fdr 0.01) with IgG input as control. Narrow MEN1 and MLL1 binding peaks were called using macs2 (parameters: -q 1e-4 --max-gap 2000 --keep-dup 1) and SICER2 (parameters: -w 100 -g 300).

Data quality

CUT&Tag reads were mapped to the human genome assembly hg38 using HISAT2 (v2.1.0). Reads mapped to ENCODE blacklist regions were excluded using the function "intersect" of bedtools (v2.27.1). Potential PCR duplicates were removed by the function "MarkDuplicates" (parameter: REMOVE_DUPLICATES=true) of Picard (v2.23.1).

Software	HISAT2 (v2.1.0)<br>featureCounts (v1.6.1)<br>Picard (v2.23.1)<br>SICER2<br>bedtools (v2.27.1)<br>deepTools (v 3.3.2)

Flow Cytometry

Plots

Confirm that:

- [x] The axis labels state the marker and fluorochrome used (e.g. CD4-FITC).
- [x] The axis scales are clearly visible. Include numbers along axes only for bottom left plot of group (a 'group' is an analysis of identical markers).
- [x] All plots are contour plots with outliers or pseudocolor plots.
- [x] A numerical value for number of cells or percentage (with statistics) is provided.

Methodology

Sample preparation	For the competitive proliferation assays using sgRNAs, the percentage of sgRNA-expressing cells (RFP+) was measured over time using flow cytometry and normalized to the starting time point (3 days after infection).
Instrument	Data were acquired on LSR Fortessa (BD).
Software	Flow cytometry data was analyzed using FlowJo software (V10).
Cell population abundance	Sorting is not relevant to this study.
Gating strategy	FSC/SCC gates were applied to determine alive cells. "Positive" population is determined by gating at the right boundary of control sample which is "negative" population.

- [x] Tick this box to confirm that a figure exemplifying the gating strategy is provided in the Supplementary Information.
